# Evaluating C-peptide Levels in Children with Type 1 Diabetes and Its Association with Anthropometric and Clinical Variables: C-peptide Levels and Clinical Variables in Pediatric T1DM

**DOI:** 10.31661/gmj.v14i.3787

**Published:** 2025-08-08

**Authors:** Raha Sahraian, Anis Amirhakimi, Parnia Kamyab

**Affiliations:** ^1^ Department of Pediatric Endocrinology and Metabolism, School of Medicine, Shiraz University of Medical Sciences, Shiraz, Iran; ^2^ Universal Scientific Education and Research Network, Fasa University of Medical Sciences, Fasa, Iran

**Keywords:** C-peptide, Type 1 Diabetes Mellitus, Insulin, HbA1c, Glycemic Control

## Abstract

**Background:**

C-peptide, a byproduct of insulin production, plays significant physiological roles, including stabilizing blood glucose levels and protecting tissues. Its potential therapeutic role in Type 1 diabetes mellitus (T1DM) management remains underexplored, particularly in preserving β-cell function and improving glycemic control.

**Materials and Methods:**

This cross-sectional study included 69 pediatric patients with T1DM at Bo Ali Diabetes Clinic, Shiraz, Iran, from December 2022 to April 2023. Patients with a disease duration less than 2 years or metabolic comorbidities were excluded. Anthropometric measurements, HbA1c, and serum C-peptide levels were collected after three months of dietary counseling and insulin adjustment. Data were analyzed using Spearman’s correlation and linear regression.

**Results:**

The study population consisted of 69 children (mean age: 11.92 ± 3.65 years; mean disease duration: 63.13 ± 33.16 months). Serum C-peptide levels (mean: 87.02 ± 73.89 pmol/L) were inversely correlated with disease duration (ρ=-0.433, P0.001) and HbA1c (ρ=-0.404, P=0.001). Regression analysis confirmed that both HbA1c and disease duration were significant predictors of C-peptide levels (P0.05). However, no significant associations were observed between C-peptide and age, weight, height, or BMI.

**Conclusion:**

Reduced C-peptide levels are correlated with poorer glycemic control (greater HbA1c) and longer disease duration in children with T1DM. These findings highlight the clinical relevance of C-peptide supplementation as a potential therapeutic strategy to preserve β-cell function and improve long-term outcomes in T1DM management. Longitudinal studies are warranted to further evaluate its efficacy.

## Introduction

Type 1 diabetes mellitus (T1DM), also known as insulin-dependent diabetes, is a persistent autoimmune metabolic disorder characterized by the loss of insulin-secreting cells in the pancreas [1]. Globally, about 30,000 individuals are diagnosed with T1DM annually, with a rising prevalence of roughly 3% per year among children [2][3]. In Iran, the prevalence of T1DM among children is estimated to be 11 per 100,000 individuals annually, with higher rates observed in urban areas [4]. Along with that, in recent years, the rising prevalence of T1DM has imposed significant healthcare and economic challenges [5]. The etiology of T1DM is multifactorial, involving genetic predisposition and environmental factors, like viral infections, although the precise cause remains unknown [6]. T1DM potentially manifests at any age, however it often exhibits two prominent peaks within the under-18 demographic: the first occurs in children aged 4 to 7 years, and the next peak arises in teenagers aged 10 to 14 years [7]. Individuals with T1DM usually experience more severe forms of metabolic dysregulation, which, according to studies, can be due to C-peptide deficiency and lead to vascular damage [8][9][10].

C-peptide is a connecting chain containing 31 amino acids synthesized by the prohormone convertase enzyme in beta cells from pro-insulin [11]. It connects the alpha and beta chains of pro-insulin, facilitating the formation of biologically active insulin [12]. In addition to being historically considered a byproduct of insulin manufacturing, a recent study has revealed the diverse physiological importance of this biomarker, including reducing inflammation, improving endothelial function, and mitigating vascular complications in diabetes [13]. It has been documented that C-peptide has multiple target organs and can exert biological effects on various tissues in the human body [14].

Studies have shown that therapeutic strategies targeting C-peptide levels, such as mesenchymal stem cell therapy and novel agents like imeglimin, improve glycemic control and beta-cell preservation [15]. Clinically, C-peptide serves as a biomarker of endogenous insulin production and is essential for the management of T1DM [16]. For instance, elevated serum C-peptide levels at diagnosis and during treatment have been associated with reduced insulin dependency and a decreased risk of diabetic ketoacidosis [17]. To study this significance, J Suh et al. [16] investigated the serum level of C-peptide in 234 children and adolescents with T1DM for a period of 15 years. The study findings indicated that patients presenting elevated serum C-peptide levels at both diagnosis and post-treatment required lower insulin doses and were at a reduced risk of developing diabetic ketoacidosis [16].

In addition, some cohort-based studies have indicated that even partial amounts of C-peptide in T1DM patients are correlated with decreased complications of diabetes including hypoglycemia and microvascular dysfunctions [18][19].

Despite these findings, the literature lacks comprehensive studies exploring the therapeutic effects of C-peptide supplementation in pediatric populations, particularly in relation to clinical-demographic factors such as age, gender, and disease duration. Our study aims to assess the relationship between serum C-peptide levels and clinical-demographic factors, including age, gender, disease duration, and metabolic parameters, in children with T1DM. By addressing these gaps, this research seeks to contribute to the development of evidence-based, individualized therapies tailored to the unique needs of pediatric T1DM patients, ultimately enhancing clinical outcomes and quality of life.

## Materials and Methods

### Study Participants and Setting

The current cross-sectional observational research was performed on 69 pediatric individuals with T1DM referred to Bo Ali Diabetes Clinic, Imam Reza Hospital, Shiraz, Iran, between December 2022 and April 2023. Participants were recruited consecutively from patients attending the diabetes clinic. The clinic specializes in pediatric diabetes care and provides multidisciplinary services, including endocrinology consultations, nutritional counseling, and psychological support. To ensure robust and reliable results, strict exclusion criteria were applied.

Individuals with a disease history of less than two years were excluded to avoid variability related to early disease dynamics, as were those with concomitant metabolic disorders such as hypothyroidism and celiac disease, which could independently influence metabolic parameters. Ethical approval was obtained from the Ethics Committee of Shiraz University of Medical Sciences (approval code: IR.SUMS.MED.REC.1401.557). We acquired written informed consent from parents or guardians, and assent was collected from participants where applicable, ensuring adherence to the rules of the Helsinki Declaration. Participant confidentiality and data security were strictly maintained throughout the study.

### Study Measurements

Participants were instructed to use the carbohydrate counting technique for insulin administration, supported by three sessions of dietary counseling over three months to ensure accuracy in carbohydrate calculation and glycemic correction [20]. These sessions included tailored guidance on portion sizes, glycemic index, carbohydrate-to-insulin ratio calculations, and strategies for managing hypoglycemia.

Adherence to dietary recommendations was monitored through self-reported dietary logs, which were reviewed during follow-up visits. Baseline anthropometric measurements, including height and weight, were recorded using calibrated equipment, and BMI was determined by dividing weight (kg) by height squared (m²). Fasting blood samples were collected in the morning at the conclusion of the three-month follow-up period and analyzed for C-peptide levels using an electrochemiluminescence immunoassay (ECLIA) with Roche Diagnostics equipment. Additional laboratory parameters, such as HbA1c, were measured through high-performance liquid chromatography (HPLC).

### Statistical Analysis

The sample size was determined using a pilot study, assuming an anticipated correlation coefficient (r) of 0.3, with a significance level (α) of 0.05 and a power (1−β) of 80%. The calculated sample size was 68 participants, rounded to 70 to account for potential dropouts. However, one participant dropped out, leaving a final sample size of 69. Data analysis was conducted by SPSS ver. 27.0 (IBM Corp., Armonk, NY, USA). The Kolmogorov-Smirnov test was performed to assess the normality of the data. he Lilliefors test was used specifically to evaluate the normality of the C-peptide variable. Quantitative data were expressed as mean ± standard deviation (SD), while qualitative data were presented as numbers (percentage). Spearman’s rank correlation was used to examine the relationship between C-peptide levels and various anthropometric and clinical factors. Scatter plots for significant associations (C-peptide vs. HbA1c and C-peptide vs. Duration) were generated using R (version 4.4.2) with the ggplot2 package. We performed multivariable linear regression with C-Peptide as the dependent variable to identify significant predictors. The assumptions of linear regression were assessed, including normality of residuals (Shapiro-Wilk test), linearity, homoscedasticity, and multicollinearity (Variance Inflation Factor, VIF). Although residuals deviated from normality (P<0.05), this was unlikely to significantly affect regression results because linear regression is robust to minor deviations from normality under large enough sample sizes. Furthermore, multicollinearity levels were acceptable (VIF<5), and residual plots confirmed no substantial violations of linearity or homoscedasticity.

## Results

**Table:** Table1. Study Participant Characteristics

**Variables (mean ± SD)**	**Total Population (n=69)**
**Age at diagnosis (years)**	11.92 ± 3.65
**Duration of disease (months)**	63.13 ± 33.16
**BMI (Kg/m²)**	18.64 ± 3.14
**Weight (Kg)**	39.58 ± 13.92
**Height (cm)**	143.12 ± 18.89
**HbA1c (%)**	9.18 ± 1.50
**C-peptide (pmol/L)**	87.02 ± 73.89
	
**Variables [n (%)]**	
**Age groups**	
**≤ 5 years**	2 (2.9)
**5-12 years**	27 (39.1)
**≥ 12 years**	40 (58.0)
**Duration of disease**	
**< 5 years**	40 (57.1)
**≥ 5 years**	29 (42.0)

Data are shown as mean ± standard deviation for continuous variables and as frequencies (percentages) for categorical ones. SD: standard deviation; BMI: body mass index.

**Table:** Table2. Correlation Analysis for C-Peptide and HbA1c

**Variable**	**Correlation Coefficient (r)**	**P-value**
**Age at diagnosis (years)**	0.063	0.607
**Duration (months)**	-0.433	<0.001*
**Weight (kg)**	0.084	0.494
**Height (cm)**	0.104	0.393
**HBA1C**	-0.404	0.001*
**BMI (kg/m²)**	-0.008	0.951

**Table:** Table3. Multivariable Regression Analysis for Predicting C-peptide

**Predictor**	**Unstandardized Coefficients (B)**	**Std. Error**	**Standardized Coefficients (Beta)**	**t**	**Sig.**	**Lower 95% CI**	**Upper 95% CI**
**(Constant)**	193.212	128.206	-	1.507	0.137	-63.069	449.492
**Sex**	8.768	15.969	0.06	0.549	0.585	-23.154	40.689
**Age**	5.364	7.373	0.265	0.727	0.47	-9.375	20.102
**Duration**	-0.832	0.278	-0.374	-2.992	0.004*	-1.388	-0.276
**Weight**	0.605	1.312	0.114	0.461	0.647	-2.942	2.646
**Height**	-0.148	1.398	-0.038	-0.106	0.916	-23.154	40.689
**HBA1C**	-14.548	5.602	-0.296	-2.597	0.012*	-25.747	-3.349

The dependent variable is C-peptide. Variables marked with * indicate statistically significant predictors (P<0.05).

**Figure-1:**
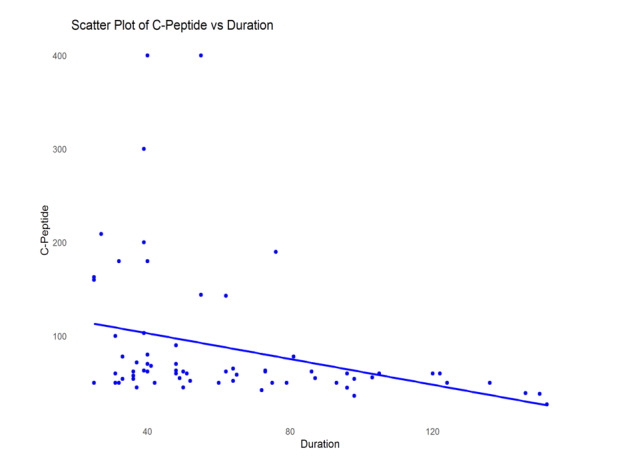
Figure 1. Scatter plot showing the association between serum C-peptide levels and disease duration.

**Figure-2:**
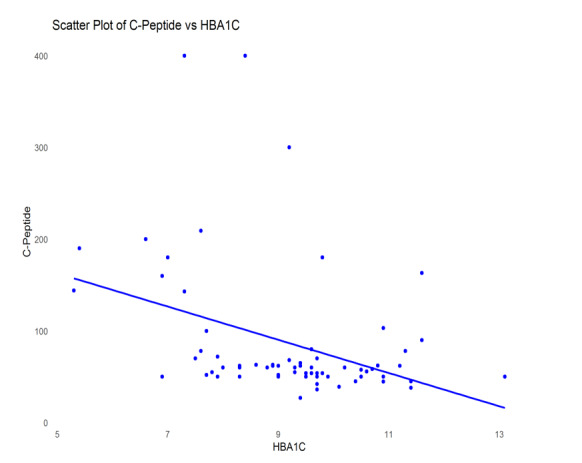
Figure 2. Scatter plot showing the association between serum C-peptide levels and HbA1c.

### Subject Characteristics

The baseline profiles of the study population are summarized in Table-1. A total of 69 children were included in this study, comprising 34 girls (49.3%) and 35 boys (50.7%). They were, on average, 11.92 ± 3.65 years old, representing a wide age range of 5 to 18 years, with 40 participants (58.0%) aged 12 years or older. The average disease duration was 63.13 months (approximately 5 years), with 42.0% of participants having a disease duration of 5 years or more. The participants had a mean weight of 39.58 ± 13.92 kg, a mean height of 143.12 ± 18.89 cm, and a mean BMI of 18.64 ± 3.14 kg/m². Laboratory evaluations showed an average serum C-peptide level of 87.02 ± 73.89 pmol/L and a mean HbA1c level of 9.18 ± 1.50%.

### C-peptide and HbA1c Levels and Comparison with Other Variables

In order to evaluate the associations between quantitative variables with C-peptide, we performed a correlation analysis. Since C-peptide did not follow a normal distribution, Spearman’s rank correlation was applied to evaluate both the strength and direction of the relationships.

The findings regarding the correlations analysis are demonstrated in Table-2. Our analysis revealed a significant negative relation between C-peptide levels and HbA1c (ρ=-0.410, P<0.001), suggesting that greater HbA1c levels were correlated with lower C-peptide levels. Similarly, a remarkable reverse association was found between C-peptide levels and disease duration (ρ=-0.424, P<0.001), suggesting that longer disease duration is linked to reduced C-peptide levels. We have created scatter plots of significant associations from this correlation (between C-Peptide and HbA1C, and C-Peptide and Duration), which are presented as Figure-1 and -2.

### Regression Analysis for Predicting C-peptide and HbA1C

A linear regression analysis was performed with C-peptide as the dependent variable and predictors age, duration, weight, height, sex, and HbA1C. Significant predictors identified were disease duration (B=-0.832, 95% CI: -1.388 to -0.276, P=0.004) and HbA1c (B=-14.548, 95% CI: -25.747 to -3.349, P=0.012), both inversely associated with C-peptide. No significant correlations between C-peptide and sex, age at diagnosis, weight, and height were observed. BMI was not included in the regression model due to including its components, height and weight, which were entered separately to avoid redundancy. Results are brought in Table-3.

## Discussion

Insulin replacement has been the primary treatment for T1DM for a long time [21]. However, it has not been effective in achieving the intended result in most cases and has not been able to fully prevent the complications associated with diabetes [21]. Multiple studies have reported that C-peptide exerts a positive influence on the functioning of different organs and tissues [22][23].

For instance, C-peptide is capable to decrease glomerular filtration rate (GFR) by 24%, enhance cardiac contractility, enhance peripheral nervous system function, and improve blood circulation in tissues [22]. As a result, C-peptide is shown to present many therapeutic impacts on the micro and macrovascular complications observed in individuals diagnosed with T1DM [14][24]. The present research was designed to evaluate the factors influencing C-peptide levels in pediatric patients with T1DM.

Our analysis did not reveal significant correlations between C-peptide levels and age, weight, height, or BMI, which is consistent with prior studies reporting weak or inconsistent associations between these anthropometric measures and β-cell function [25][26]. his suggests that residual β-cell function in T1DM is influenced more by metabolic, genetic, and immune factors than by physical growth parameters.

In contrast, factors such as metabolic stress, insulin sensitivity, and immune modulation may have a more substantial impact on residual β-cell function. For instance, genetic evidence has recommended that variations in specific loci related to β-cell survival, inflammation, and glycemic regulation play a pivotal role in shaping β-cell dynamics, irrespective of anthropometric measures [27]. Moreover, the multifactorial determinants of β-cell preservation emphasize the need for a more comprehensive approach that includes evaluating immune markers, autoantibody profiles, and metabolic stress indicators.

Another notable observation from earlier studies is the potential interaction between anthropometric measures and environmental or hormonal factors during critical periods of growth. Although anthropometric measures may not directly correlate with C-peptide levels, they may influence other variables, such as growth-related hormones or adipokines, that indirectly affect β-cell function [28]. Therefore, a deeper understanding of how these secondary factors interact with β-cell function is necessary for improving disease outcomes in T1DM.

Moreover, we observed a significant negative correlation between C-peptide levels and disease duration, which was confirmed by multivariable linear regression analysis. Our regression analysis confirmed that disease duration was a significant predictor of declining C-peptide levels, which aligns with studies reporting that C-peptide levels decrease rapidly in the initial years following diagnosis and decline more gradually in later stages [29][30].

The observed pattern is consistent with previous research, which found that C-peptide levels reduce rapidly in the first years following diagnosis, followed by a slower rate of decline in the later stages [30][31].

This decline highlights the importance of early intervention strategies to maintain β-cell function, particularly in the early phases of T1DM.

Another meaningful result was the negative association between C-peptide levels and HbA1c, suggesting that elevated HbA1c levels were related to lower C-peptide levels. This finding underscores the functional impact of residual β-cell in glycemic control. C-peptide, a byproduct of insulin secretion, is a reliable marker of endogenous insulin production. As β-cell function declines over the progression of T1DM, lower C-peptide levels are typically accompanied by poor glycemic control, as reflected by elevated HbA1c values [32][33]. This finding corroborates prior studies that associated maintained C-peptide levels with improved HbA1c and reduced risk of complications [34][35].

These findings suggest that therapies aimed at preserving β-cell function, such as immunomodulatory treatments, may have a significant impact on long-term metabolic control and patient outcomes [36]. The link between higher C-peptide levels and improved glycemic outcomes, as reflected by lower HbA1c levels, emphasizes the potential therapeutic benefit of C-peptide supplementation in managing T1DM [37].

Although insulin replacement therapy is essential for managing T1DM, it is often insufficient in completely reducing the risk of complications and attaining ideal control of blood sugar levels [38].

On the other side, C-peptide has been identified as a biologically active peptide that has several effects, providing protection to different organs and tissues such as the kidneys, heart, and nervous system [39].

The mechanisms responsible for the important benefits of C-peptide on glycemic control are diverse. Firstly, studies demonstrated that C-peptide may enhance insulin sensitivity and the use of glucose in peripheral tissues, leading to an enhancement in overall glucose homeostasis [40].

In addition, C-peptide has vasodilatory properties, which increase blood flow to tissues that are sensitive to insulin and promote the administration and effectiveness of insulin [41]. Moreover, C-peptide has been linked to modulating inflammatory pathways and oxidative stress, both of which play a role in the onset of insulin resistance and β-cell impairment in T1DM [41]. By specifically addressing these abnormal physiological processes, the addition of C-peptide may provide a potentially effective supplementary treatment to insulin replacement therapy, especially for those who still have some remaining β-cell function.

This study has several strengths. By focusing exclusively on pediatric patients, it provides valuable insights into the early course of T1DM and the role of C-peptide during a critical developmental period. Additionally, the use of clinically relevant parameters, such as HbA1c and growth metrics, enhances the practical applicability of the findings.

However, there are limitations to consider. The cross-sectional design precludes causal inferences regarding the observed relationships. The modest sample size, while sufficient for initial analyses, may limit the generalizability of the findings to larger populations. Furthermore, we did not control for potential confounding factors, such as differences in insulin regimens, dietary habits, or autoantibody profiles, which may have influenced the results.

Future research should focus on longitudinal studies to better understand the temporal dynamics of C-peptide decline and its association with glycemic control and complications. Specific areas of investigation could include the role of genetic and immune factors in preserving β-cell function, the impact of C-peptide supplementation on vascular and neural complications, and the effects of lifestyle interventions on C-peptide dynamics. Exploring these aspects will help design personalized therapies to optimize outcomes in pediatric patients with T1DM.

## Conclusion

This study demonstrates significant negative correlations between C-peptide levels and both HbA1c and disease duration in children with T1DM, highlighting the critical role of residual β-cell function in glycemic control. These findings underscore the potential of early interventions, including C-peptide supplementation, to preserve β-cell function, improve glycemic stability, and reduce complications. Future research should focus on longitudinal studies and personalized treatment strategies to optimize pediatric T1DM management and explore the integration of C-peptide into existing therapeutic protocols.

## Conflict of Interest

The authors declare that they have no conflict of interest.
